# Supplementary material for: Are carbon emissions trading and green financial instruments synergistic? -Comprehensive quantitative research based on content analysis
Source: PLoS One. 2024 Mar 7;19(3):e0298601. doi: 10.1371/journal.pone.0298601 (PMC10919601; doi:10.1371/journal.pone.0298601)
Supplement: S1 Data — (ZIP) [file pone.0298601.s001.zip › Data/地级市Z_F工作报告.zip!/╣π╢1⁄2/╤⌠╜¡╩╨/└┤╫╘╣1⁄2╓┌║┼í╛┬φ┐╦╩2╛▌═°í┐_2012.docx]

各位代表：

　　现在，我代表市人民政府向大会作工作报告，请予审议，并请市政协委员和其他列席人员提出意见。

　　科学发展结硕果

　　2011年，是阳江“在加快发展中转变方式、实现幸福追赶”征程上开拓奋进、硕果累累的一年。一年来，在上级党委、政府和市委的正确领导下，在市人大及其常委会和市政协的监督支持下，市政府团结带领全市人民，深入贯彻落实科学发展观，攻坚克难谋跨越，开拓创新干实事，着力提速度、转方式、优环境、强管理、惠民生，经济社会在新起点上取得新进步，实现了“十二五”良好开局，谱写了阳江科学发展史上新的华章！

　　——经济实力快速跃升。预计全市实现地区生产总值779亿元，增长16%；人均生产总值32000元，增长15%；地方财政一般预算收入34.95亿元，增长37.7%；固定资产投资408.5亿元，增长43%；外贸进出口总额21.6亿美元，增长20%，其中出口19.3亿美元，增长20%；社会消费品零售总额438.9亿元，增长18%。主要经济指标增速均居全省前列。

　　——产业转型升级加快。工业向适度重型化转变。规模以上工业总产值858亿元，增加值219亿元，分别增长30%和30.5%。重工业增长36%，比轻工业快13个百分点。广青镍合金一期、华润阳春水泥一期等大项目顺利投产，电力能源、节能玻璃、汽车空调等项目稳步推进。农业向集约化发展。全市特色经济作物291.1万亩，新建成3个面积共3000亩的现代农业园区。培育发展了一批国家和省级重点农业龙头企业，2家企业被授予全国农产品加工示范基地。农机装备及作业水平明显提高，水稻耕种收综合机械化水平达60.3%。海洋渔业蓬勃发展。渔业总产值107.3亿元，增长7.1%，水产品总产量102万吨，增长4.1%。“中国南海渔都”落户阳江，海陵岛入选首批国家海洋公园。服务业向高端化迈进。阳江被省确定为旅游综合改革示范市。十里银滩综合开发等旅游项目建设进展顺利。全年接待游客总人数899万人次，旅游总收入66.3亿元，分别增长23.7%和55.3%。商贸流通新型业态加快发展。新都汇时代广场、华科国际家居生活广场等专业市场项目相继动工。自主创新能力不断增强。国家刀剪及日用金属工具质量监督检验中心顺利建成；实施科技项目31项；新增高新技术企业1家；1-10月获得授权国家专利681件，增长9.9%。

　　——城乡建设扎实推进。交通等基础设施进一步完善。阳江港11、12号码头试产，13、14号码头加快建设，开阳高速公路阳江立交及连接线工程即将建成，阳阳高速至海陵大堤连接线等项目动工建设，广东西部沿海铁路阳江段等项目前期工作扎实推进；市区防洪工程首期基本完工，27宗中小型水库除险加固工程和2010年“9•21”特大洪灾水毁工程、耕地复垦等项目全部竣工。市政配套设施日臻完善。西平路、东门路、二环路等市政道路，鸳鸯湖景区、金山植物公园等公共场所整治改造工程顺利完成。高新区污水处理厂、阳西县生活垃圾填埋场等一批环保设施建成使用。宜居村镇“八个一”创建活动深入开展，300人以上自然村道路硬底化改造任务全面完成，涌现出一批生态、文明示范村镇。

　　——群众生活持续改善。预计全市城镇居民人均可支配收入16837元，增长15%；农民人均纯收入7687元，增长15.5%。十件民生实事圆满完成。社会保险体系逐步建立，新型农村养老保险参保人数40.7万人。城乡医保一体化扎实推进，参保人数220万人，覆盖率达100%。纳入低保救助的困难家庭3.6万户8.3万人，实现了应保尽保。“稳价惠民工程”大力实施，建成平价商店52家，向城乡困难群众发放临时价格补贴1100万元。扶贫开发“双到”工作纵深推进，170条贫困村投入帮扶资金5.78亿元，6300户贫困户、2.39万人实现稳定脱贫，扶持贫困村发展集体经济项目416个，村集体经济年收入全部达3万元以上。劳动力培训转移成效明显，培训3.99万人次，新增转移劳动力7.45万人，城镇登记失业率持续下降至2.45%。

　　——社会事业全面进步。各类教育均衡发展。学前教育毛入学率84.3%，初中三年保留率92.3%，高中阶段教育毛入学率96.1%；中职教育规模和质量有效提升，普职比52∶48；高等教育内涵建设进一步强化，办学质量有新提高。医药卫生体制改革深入推进，48间政府办基层卫生机构全面实施国家基本药物制度，人均药品费用下降28.3%。市中医院新院、市公共卫生医院建成投入使用，10间镇级卫生院标准化建设和9间社区卫生服务中心改造全部完成。区域卫生信息化建设走在全省前列。市体育运动学校、市帆板基地、市美术馆、市非物质文化遗产展示馆和422家农家书屋顺利建成。《阳江市志》正式出版。圆满完成第六次全国人口普查任务。人口计生服务管理水平不断提高，人口自然增长率控制在7.5‰以内，荣获“全国人口计生综合改革示范市”称号。广播电视、新闻出版、食品药品、民族宗教、外事侨务、统计、审计、打私、打假、气象、地震、科普、档案、人防、双拥、武装、国防动员等工作取得了新成效，妇女、儿童、青少年、老龄、关工委、老促会、红十字、慈善、残疾人等事业实现了新发展。

　　——和谐局面有效巩固。依法接受市人大及其常委会的工作监督和法律监督，自觉接受政协的民主监督，认真办理人大代表议案6件、建议64件，政协议案1件、提案178件，办复率均达100%。机关作风整顿工作深入开展，对全市77个单位进行社会满意度测评。政府管理不断创新，精简高效、运转协调、行为规范的行政管理体系逐步建立完善。社会管理有效加强，重大事项社会稳定风险评估全面实施，社会治安综合治理扎实开展，荣获“平安城市”和“中国社会治安综合治理模范城市”称号。群众工作日益深化，新增社会组织50个，在全省率先全面开通网络问政平台。应急管理体系和工作机制逐步健全，公共安全和处置突发事件能力明显提高。安全生产“一岗双责”有效落实，没有发生重特大安全事故。

　　各位代表！

　　2011年取得的不凡业绩，为本届政府工作画上了圆满句号。回顾过去的五年，我们始终坚持以科学发展观统领全局，整合资源办大事、谋划长远抓项目、理顺机制提效率、精细管理上水平，出色完成了“十一五”规划和市五届人大历次会议提出的目标任务。

　　过去的五年，是攻坚克难、实干巧干，科学发展阔步向前的五年。我们认真贯彻落实国家、省一系列重大决策部署，不断探索完善新时期阳江经济社会发展的新思路新举措，坚定信心应对挑战，快马扬鞭推进发展，演绎了迎难而上、跨越发展的壮美乐章。五年来，全市地区生产总值、人均生产总值、地方财政一般预算收入、固定资产投资、外贸出口年均分别增长14.1%、13.3%、28.8%、35.7%和14.5%，其中人均生产总值在粤东西北地区率先突破5000美元，成为后发地区追赶发展中名副其实的“快马”。面对国际金融危机的冲击，我们在上项目、促消费、扶企业、扩出口等方面打出了漂亮的“组合拳”，投资、消费、出口“三驾马车”在困难时期均实现逆势飘红。面对工业基础薄弱等突出问题，我们加大借力发展力度，加快产业转移园区建设，积极承接产业转移，园区规模不断扩大，经济效益日益凸显，在全省产业转移园区三次竞争评选中均取得优异成绩；联合省有关部门在香港、上海、东莞等地举办大型投资环境推介会，成功引进了明阳风电设备制造等一批大企业大项目，实际利用外资连年大幅增长。面对“黑格比”、“凡亚比”等重大自然灾害，我们科学预判、周密部署，众志成城开展抗灾复产，把灾害损失降到最低，以最短时间夺取了重建家园的重大胜利。通过五年的努力，初步走出了一条后发地区追赶发展的新路子，在全省贯彻落实科学发展观考核中，由2009年的第16名上升到2010年的第11名。

　　过去的五年，是加快转型、调优结构，现代产业体系提速建设的五年。我们以调整优化产业结构为着力点，加快转变经济发展方式，全力推进重大项目建设，积极构建现代产业体系。为做大做强工业，我们加快工业适度重型化，坚持培育本土企业与招商引资并举，努力推动工业经济提质增效，以阳江核电站、阳西电厂、沿海风电等为支撑的电力能源基地建设全面推进，以广青镍合金为代表的新兴产业提速发展，钢铁、水泥、五金刀剪等传统产业加快转型。轻重工业比例由2006年61∶39调整为2011年53∶47，规模以上工业总产值和增加值年均分别增长24.9%和25.5%。为巩固农业的基础地位，我们在不断提高粮食安全保障水平的同时，着力发展效益农业，水果、南药等特色农业规模新增33.6万亩，农业总产值年均增长3.6%。为建设海洋经济强市，我们充分发挥资源优势，全面发展海洋经济，推动海洋渔业转型升级，促进了临港工业、滨海旅游、海洋文化等产业发展，海洋经济总产值由2006年86.4亿元增加到2011年282亿元。为繁荣发展以旅游业为龙头的现代服务业，我们以打造国际休闲旅游度假胜地为目标，科学整合开发旅游资源，加强旅游环境整治，引进了南湖国旅等一批大企业，开发建设了一批旅游大项目和高星级酒店，成功创建为“中国温泉之乡”，初步塑造了“浪漫银滩、宋船古韵、温泉之都、水墨阳江、休闲绿城”旅游品牌。通过五年的努力，全市产业结构不断优化，产业比例由2006年28∶37.9∶34.1调整为2011年19.5∶45.9∶34.6，三次产业协调发展的新型工业化城市加速崛起。

　　过去的五年，是加快建设、强化管理，城乡面貌变化巨大的五年。我们以建设“让本地人自豪、令外地人羡慕”的城市为目标，整合资源，科学运营，统筹城乡协调发展，努力建设宜居乐居城市。强化规划引领，完成了新一轮土地利用总体规划和城市总体规划修编，启动了市区与阳东县东城镇、雅韶镇、红丰镇一体化。重视基础建设，建成了阳云高速阳阳段、阳江港4个万吨级以上码头等一批重要交通设施，公共交通体系进一步完善。加快了城南新区和中洲新城等重点区域的规划建设，高标准改造建设了东风路、江朗大道等一批市政道路，完善了一批公园、绿道和污水垃圾处理等设施，推进了市区“一河两岸”改造工程，城市框架不断拓展，城市功能逐步优化。理顺了市区环境卫生管理体制。深入开展创建宜居城乡活动，加强镇村规划和综合治理，创建了11个宜居示范镇和69个宜居示范村。加大生态建设和环境保护力度，水环境明显好转，空气质量持续改善，连续七年在全省环保考核中被评为优良。坚持节约集约用地，实施了名扬国际广场等一批“三旧”改造项目，五年来新增建设用地规模42.33平方公里，连续12年实现耕地占补平衡。通过五年的努力，我市城市品位大幅提升，城乡一体化明显加快，镇村面貌更加靓丽，成功创建为省级园林城市。

　　过去的五年，是以人为本、执政为民，民生福利大幅改善的五年。我们坚持把保障和改善民生作为建设幸福阳江的出发点和落脚点，致力听民声、重民意、解民困、维民利，每年民生支出占地方财政一般预算支出的比例达56%以上，老百姓密切关注的教育、医疗、治安等情况明显改善。大力发展教育事业。在巩固发展九年义务教育的基础上，投入6亿多元迁建了市一中和市一职中，新建和扩建了阳春一中、阳东二中、阳西二中等20多所学校，高中阶段教育毛入学率大幅提高了50.3个百分点，高考成绩稳步提高，成为欠发达地区教育事业跨越发展的新范本。加快构建覆盖城乡的基本公共文化服务体系，广东海上丝绸之路博物馆、市水上活动中心和市文化艺术中心等一批文化体育设施建成投入使用。建立完善覆盖城乡的社会保障体系，城镇职工“五大险种”参保人数由2006年72.5万人次提高到2011年104.9万人次；农村年人均收入低于1500元的困难家庭全部纳入低保范围。积极统筹资源，妥善解决了市直困难企业和医疗机构1.3万人历史遗留的社保问题。切实加强培训就业，五年来参加职业技能培训19.3万人，其中免费培训农村劳动力13.4万人。高度重视敬老院建设，积极实施“千间敬老福星工程”，投入资金7000多万元，完成改扩建敬老院48所。狠抓扶贫开发“双到”工作，170条贫困村有劳动能力的贫困户脱贫率达95%。认真落实渔业柴油涨价补贴政策，发放补贴12.5亿元。着力解决住房难问题，建成廉租住房1232套，2009年和2010年住房保障工作被省评为优秀；改造农村泥砖住房16834户，安置渔民上岸居住686户。大力推进农村饮水工程改造，解决了69万人饮水安全问题。通过五年的努力，我市人民群众生活水平明显改善，城镇居民人均可支配收入、农民人均纯收入年均分别增长10.5%和10.9%，人民群众幸福指数大幅提升。2010年阳江入选“中国十大最具幸福感城市”。

　　过去的五年，是开拓进取、改革创新，发展活力迸发的五年。我们积极借鉴发达地区特别是珠三角的经验，勇闯新路，破解难题，不断推进改革创新，有效释放跨越发展的内在动力。大刀阔斧地进行市县两级政府大部制改革，市政府工作部门在全省地级市中机构最为精干，在全省政府机构改革评估中得分居首。完成了高新区区镇港合一、海陵区扁平化管理和岗侨区“三融入”改革。整合了市公共资源交易平台，行政效能有效提高。着力深化行政审批制度和简政强镇改革，共减少行政审批事项156项，市级设置的行政审批事项全部取消，市直单位行政处罚自由裁量权得到了量化细化。建立和完善土地储备制度，促进土地资源合理配置，优化了土地供应布局和结构。整合优质国有企业，组建了市公用事业集团，基本解决了困难国企改革等问题。完善公务员和事业单位录用招考制度，干部队伍素质明显提高。通过五年的努力，我市推动科学发展的体制机制进一步完善，市场经济活力进一步激发，社会发展动力进一步增强。

　　过去的五年，是依法施政、提升服务，发展环境实现嬗变的五年。我们在政府系统推进规范化建设和精细化管理，出台了一系列规章制度管人、管事、管财。全力推进依法治市，“五五”普法任务全面完成，“六五”普法扎实开展，全民法律意识进一步增强。制订《阳江市人民政府行政决策程序规定》等规章制度，建立公众参与、专家论证、政府决定相结合的行政决策机制，保证了政府规范管理、依法行政、高效廉洁。完善市行政集中服务大厅建设，市直部门234项审批服务项目进驻，实现了行政服务承诺时间比法定时间、办结时间比承诺时间“双减半”。加强机关作风整顿，每年开展企业评议涉企政府部门、工业企业集中服务月等活动，投资环境明显改善。加大行政监察和审计力度，政府系统廉政建设进一步加强。实施人才发展战略，人尽其才、充满活力的用人机制逐步形成。推进文化名城“五个十”工程建设，城乡文明程度不断提高。创新社会管理，鼓励社会组织和企业参与提供公共服务，公共服务能力和效率持续提高。高度重视社会治安管理，重拳打击各种违法犯罪活动，社会治安实现了根本性好转，群众安全感显著增强。在全省率先成立三级综治信访维稳中心，妥善解决了一批社会矛盾纠纷问题，非正常上访逐年实现降量退位。通过五年的努力，我市政务环境、法治环境、投资环境、人文环境全面改善，齐心协力谋发展的浓厚氛围在漠阳大地蔚然形成。

　　各位代表！

　　发展的成绩令人鼓舞，奋斗的历程让人铭记。五年来，我们在困难中经受了考验，在发展中凝聚了民心，在实干中创造了佳绩。五年来，阳江从经济到社会、从城市到乡村均发生了深刻变化，得到了各级领导、社会各界和人民群众的充分肯定和广泛认同。这离不开上级党委、政府和市委的坚强领导，离不开市人大、市政协与各民主党派的监督支持，离不开全市人民、外来投资者以及驻阳江部队、武警官兵的共同努力，离不开港澳台同胞、海外侨胞以及国际友人的关心帮助。在此，我代表市人民政府，向所有为阳江建设和发展作出贡献的同志们、朋友们，表示衷心的感谢并致以崇高的敬意！

　　五年的发展启示良多，积累的经验弥足珍贵。我们深切体会到：要实现科学发展、幸福追赶，必须坚持以经济建设为中心，千方百计做大经济总量，促进经济结构优化升级，推动经济社会又好又快发展；必须坚持解放思想，推进体制改革和制度创新，创造性地开展工作，激发加快发展的强大动力和活力；必须坚持发展经济与保护资源环境相结合，加强生态环境保护，建设人与自然和谐相处的人居环境；必须坚持以人为本，下大力气解决人民群众最关心、最直接、最现实的利益问题，实现幸福社会的共建共享；必须坚持依法行政，转变政府职能，切实提高政府善政善治水平，营造良好的发展环境。

　　成绩固然可喜，问题尤需警醒。当前我市经济社会还存在不少问题：经济总量小、产业转型升级任务艰巨；利用外资质量有待提升，具有支撑作用的优势产业、规模企业和重大项目不多；群众生活水平和富裕程度还不高，民生改善的力度还需进一步加大；城乡建设基础较差，市政设施不足和配套不完善；历史遗留问题和新的社会矛盾交织，社会管理难度增大；部分政府工作人员的综合素质未能适应新形势的要求，行政服务水平、管理能力和办事效率有待进一步提高等。对此，我们将采取有效措施，切实加以解决。

　　幸福追赶新愿景

　　今后五年，阳江正处于转型崛起的黄金机遇期。虽然世界经济不稳定的因素很多，但我国经济稳健发展的基本面和长期趋势没有改变。《广东海洋经济综合试验区发展规划》上升为国家战略，为海洋资源丰富的阳江拓展了更大的发展空间。粤西沿海铁路和港珠澳大桥的兴建，更加密切了我市与珠三角核心区的联系。经过建市20多年的发展，我市综合实力大幅提升，基础设施更加完善，内生动力显著增强，全市上下团结一心，干部群众精神振奋，为经济社会又好又快发展奠定了坚实基础。需要直面的是，未来五年也是挑战最多、最容易落入“中等收入陷阱”的危险期。随着经济的发展，环境、资源等约束的压力不断加大，土地、劳动力、能源等生产要素供给更加紧张；随着社会结构转型加快，人民群众多层次、多样化的需求越来越高，社会建设和公共管理的任务更加繁重；随着区域之间竞争日趋激烈，我们正面临着“标兵更远，追兵更近”的严峻形势，抢先一步是跨越，停顿一步就掉队，确保不落伍更加艰难。面对复杂多变的形势，我们要切实增强使命感、危机感和紧迫感，借天时、扬地利、聚人和，站在新的历史起点上，努力实现后发快进、跨越发展。

　　对今后五年的发展，市第六次党代会已描绘了宏伟蓝图。市政府将全面贯彻落实科学发展观，按照市第六次党代会的战略部署，紧紧围绕“在加快发展中转变方式、实现幸福追赶”的核心任务，深入实施“十二五”发展规划，坚持赶超为先、发展为重，切实增强经济综合实力，保障和改善民生，维护社会和谐稳定，努力打造三次产业协调发展的新型工业化城市，再创幸福追赶的新辉煌！

　　——奋干五年，让经济更加繁荣。到2016年，全市地区生产总值和地方财政一般预算收入分别突破1580亿元和73亿元，年均分别增长15%和18%，阳江成为实力明显增强、产业特色鲜明、发展充满活力的后起之秀。工业实现历史性跨越。各大转移园区成为新的产业高地，以阳江核电为龙头的电力能源基地、以广青镍合金为龙头的镍合金产业基地、以华润水泥为龙头的新型建材生产基地、以临港工业区为龙头的海洋经济特色产业基地相继建成，一批产值超百亿元的大企业脱颖而出，一批产值超十亿元的企业勇立潮头。高效农业规模进一步扩大。10多个万亩以上的特色农业基地建成，实现千亩成块、万亩连片。海洋经济强市加速构建，现代渔业走在全国前列。旅游突破发展。海陵岛以十里银滩为核心的高端旅游度假区基本建成，力争成为全省黄金海岸旅游品牌核心区；喀斯特地貌、温泉资源等品牌在全省打响；乡村游、山水游、休闲度假游、运动保健游等旅游业态设施进一步完善，接待能力倍增，旅游人次和旅游总收入均实现翻番，成为国内外著名的休闲旅游度假胜地。

　　——奋干五年，让民生更加普惠。到2016年，城镇居民收入实现快速增长，农村群众生活全面改善，城镇居民人均可支配收入和农民人均纯收入分别达到2.7万元和1.27万元，年均分别增长10.7%和10.5%以上。社保体系覆盖城乡，住房保障、城乡低保、农民养老保险、农村五保户供养和特困群众救助等制度不断健全，人人享有基本生活保障。新一轮教育布局调整顺利完成，两阳中学新校区建成使用，青少年均享有公平的教育机会。市养老中心、市人民医院新住院大楼和市妇幼保健院新院等一批工程相继建成，覆盖全市的医疗服务网络和区域卫生信息化更加完善，基本实现群众“小病不出村、常见病不出镇、大病不出市”。

　　——奋干五年，让城乡更加秀美。到2016年，市区建成区面积达到57平方公里，力争创建成为国家环保模范城、国家园林城市和国家卫生城市。通过统筹推进市区、县城、小城镇和新农村建设，形成区域协调联动、城乡一体化发展的新格局。把城南新区建设成为中央商务区，市区“一河两岸”打造成为城市新亮点，一批公园、广场和绿道建成使用，现代综合交通运输体系进一步完善，交通出行畅通有序，市区“净、绿、亮、美、畅、宁”水平全面提升，县城功能不断完善，镇村环境干净整洁。生态环境保持良好，努力做到水更清、天更蓝、山更绿、空气更清新。

　　——奋干五年，让文化更加昌盛。公共文化服务体系逐步健全，基层文化设施覆盖工程全面实施，国家海洋公园、广东海洋历史博物馆和市演艺中心等文化设施基本建成。“南海Ⅰ号”相配套的文化产业不断发展，刀剪、风筝、漆艺等传统特色文化产业进一步做大做强。社会主义核心价值体系建设有效推进，社会公德、职业道德、家庭美德和个人品德教育深入开展，市民素质和文明程度有效提高，新闻媒体舆论引导能力明显增强，文化生活更加丰富多彩，文化软实力大幅提升。

　　——奋干五年，让社会更加和谐。民主法制进一步完善，群众权益得到切实保障，公民政治参与有序扩大，社会公平正义得到有效维护。社会服务管理体系逐步健全，公共产品的供给和质量稳步提高，城乡之间、不同社会群体之间享受公共服务的差距明显缩小。群众工作持续加强，群众诉求渠道畅通规范，社会治安打防控体系进一步完善，社会管理工作走在全省前列。

　　各位代表!

　　未来五年，将是阳江经济大发展、城乡面貌大变化、公共服务大提升的五年。实现宏伟目标，需要凝聚全市上下的智慧和力量。我们必须以奋发图强的志气、争创一流的勇气、攻坚克难的锐气，奋力谱写阳江幸福追赶的新诗篇，把绚丽的蓝图化为美好的现实。我们坚信，经过全市人民的共同努力，阳江这片热土，必将在科学发展的康庄大道上取得新的更大成绩，一个富裕、惠民、文明、和谐、宜居、活力的幸福阳江必将屹立在南海之滨！

　　务实创新抓开局

　　2012年，是新一届政府开局之年，也是“十二五”规划全面实施的关键一年。今年全市经济社会发展的主要预期目标是：地区生产总值增长15%，规模以上工业总产值增长23%，规模以上工业增加值增长24%，农业总产值增长4.5%，地方财政一般预算收入增长18%，固定资产投资增长20%，社会消费品零售总额增长16%，外贸进出口总额增长13%，实际利用外资增长15%，城镇居民人均可支配收入和农村居民人均纯收入分别增长12%，城镇登记失业率控制在3%以内，人口自然增长率控制在8.5‰以内，居民消费价格涨幅控制在4%左右，单位生产总值能耗控制在省下达指标范围内。

　　实现上述目标，要重点抓好以下工作：

　　一、以调优结构为主线，努力提升经济发展速度和质量

　　坚持把调结构、转方式作为经济工作的主线，加快培育特色明显、富有潜力的产业支撑体系，努力提升经济发展速度和质量。

　　切实壮大工业规模。着力抓好重点工业项目，加快阳江核电站和阳西电厂3、4号机组建设，力促阳江抽水蓄能电站、海螺水泥等项目上马，争取世纪青山镍合金、嘉吉粮油二期等项目尽快投产。加快区域创新体系建设，扶持新建一批工程技术研发中心和重点实验室。提高企业自主创新能力，开展特色优势行业科技提升行动。大力实施技术标准战略，充分发挥国家刀剪及日用金属工具质量监督检验中心的作用，促进五金刀剪产业转型升级。

　　大力发展现代农业。在稳定粮食生产的基础上，加快建设漠阳江两岸水果产业带，大力发展花卉、南药、蚕桑等优质高效农业。加强农业品牌建设，力争年内新增省级农业标准化示范区2个、省级名牌农产品3个以上。积极引进培育农业龙头企业，发展农产品精深加工。以广东建设海洋经济综合试验区为契机，优化发展海洋渔业，着力建设跨洋性远洋渔业基地，巩固提高远洋捕捞业。加快青洲岛和南鹏岛深水网箱产业园区建设，打造一批健康养殖示范场。创建溪头国家一级渔港、对岸区域性渔港，促进渔港全面升级。

　　搞活做旺以旅游为龙头的第三产业。优先发展旅游业，力争将我市纳入全省黄金海岸海滨旅游规划，以海陵岛为龙头，努力打造滨海旅游开发示范区。加快十里银滩、凤凰湖温泉度假区、月亮湾等景区景点和一批高星级酒店等旅游配套设施建设，提高旅游接待能力。支持沙扒、东平、春湾等镇建设成为具有独特风情的旅游美镇。发挥“南海Ⅰ号”品牌效应，不断提升旅游的知名度和美誉度。大力发展现代物流业，重点推进阳江五金刀剪商贸城、汽车商贸城、新都汇时代广场、华科国际家居生活广场、万丰工业原料城等物流基地建设。改造提升商贸餐饮等服务业，加快发展商务会展、金融、通信、康体等新兴服务业。

　　二、以扩量提质为目标，努力加快招商引资步伐

　　坚持把招商引资作为加快经济发展的主要抓手，完善招商政策，强化奖补措施，创新招商方式，不断提升招商引资水平。

　　突出招引大项目。以“扩张总量、提升质量”为目标，强化招商力量，突出招商重点，拼抢大项目，把目光瞄准世界500强和中央、省属国有企业，跨国集团和国内上市公司，全面开展招大引强突击战，着力引进一批带动能力强、产业关联度高、集聚效应明显的项目，努力提高招商引资的质量和效益。

　　突出招引临港工业项目。依托海陵湾岸线资源和阳江港等优势，着力招引一批重化项目落户，力争在重型装备、有色金属、精细化工、粮油加工、水产品加工等领域实现突破，构筑临港工业基地。加快国华电力煤电一体化等项目前期工作，力促蓝袍湾、白沙湾风电场等项目动工。

　　突出扩大外贸出口。加快转变外贸发展方式，积极创建国家级餐厨用品五金刀剪外贸转型升级示范基地，扩大自主品牌和高附加值产品出口。鼓励企业参加国际知名展会，支持更多企业取得进出口经营权，大力开拓国际市场。帮助企业稳妥应对各种困难和贸易摩擦，用好国家鼓励出口政策，确保外贸出口稳定增长。完善阳江口岸联检综合大楼配套设施，开展共建文明口岸活动，进一步提高通关效率，构建便捷高效的“绿色通道”。

　　三、以完善园区建设为依托，努力提高“双转移”水平

　　产业转移园区是加快发展的主阵地和重要载体。要集中力量，加大投入，创新机制，重点突破，推动园区建设，确保“双转移”工作取得实效，打造全省“双转移”示范基地。

　　提升园区建设水平。按照“一业主导、多业协作”的思路，完善规划、强化功能、优化布局、突出特色，把园区建设成为宜工、宜商、宜居、宜业的乐土。在完善水、电、路等基础设施的同时，加快建设物流、信息、配送、污水处理、生活配套、公共服务等设施，切实增强园区的承载、孵育和服务功能。创新和完善园区管理机制，拓宽投入渠道，发挥省市专项扶持资金的导向作用，鼓励和引导社会资金参与园区建设。适当提高项目入园门槛，规范项目用地管理，合理确定投资强度，实现节约集约用地。加强与珠三角合作方政府以及商会的沟通联系，积极引导企业和项目向园区转移，壮大园区规模，提升园区档次。

　　提升劳动力转移就业水平。大力实施全民技能提升储备计划，加快培养一批市场急需的中高级技工和熟练工人，着力提升本土人力资源竞争力。加强各类就业培训载体建设，完善“校企合作”等培训模式，深化与珠三角地区劳务合作，促进农村劳动力向本地重点企业、工业园区和珠三角等发达地区转移就业。

　　四、以加快项目建设为重点，努力完善基础设施

　　发展要提速，基础设施须先行。要进一步加快完善交通、水利、电力等基础设施，为跨越发展营造良好环境。

　　加快一批交通项目建设。快速推进阳云高速罗阳段、325国道改造等项目建设，力促广东西部沿海铁路阳江段动工。加快省道、县道以及镇村公路建设和升级改造，实施海陵大堤拓宽工程。推动阳江港5-10万吨码头和10万吨级航道疏浚工程建设，建成一批深水泊位和专用码头，打造地区性重要港口。

　　加快一批水利项目建设。抓住国家和省大兴水利的机遇，全面实施海堤加固达标工程，重点抓好四围联围等水利工程和儒洞河、那龙河、潭水河等流域治理工程建设，提高防洪排涝保安能力。加快病险水库除险加固和灌区改造步伐，推进北河水库、沙湾水库和双捷水利枢纽改造工程建设。抓好城乡供水设施建设，完善应急供水系统，动工建设市第二水厂和海陵岛水厂。推进农村饮水安全工程，通过扩网、改造、联通、整合和新建等措施，在县域内形成高保证率和统一供水标准的农村供水网络。

　　加快一批电力和通讯项目建设。实施农村电网改造升级工程，年内完成110千伏市区石湾站等一批电力项目，建设500千伏阳江核电接入系统工程阳江段，为发展提供可靠的电力保障。完善第三代无线网等通讯项目建设，加快无线城市发展步伐。

　　五、以建设宜居城乡为载体，努力加速城市化进程

　　突出宜居和可持续发展理念，坚持以规划为龙头，以人口聚集为主攻方向，以增强城市服务功能、改善人居环境为着力点，推进新型城市化进程，加快建成宜居乐居滨海新城。

　　以更高起点修编城乡规划。加快修编中心城区近期建设规划和综合交通、管网、绿化、商业网点、公益设施布局等专项规划。加强城郊区域与中心城区规划衔接，重点抓好城南新区、中洲新城、海陵岛的规划建设，推进市区与阳东县东城镇、雅韶镇、红丰镇一体化，构建以城带乡、城乡共融的城镇群发展格局。

　　以更高标准推进城市建设管理。抓好南浦大道等一批市政道路和市区“一河两岸”工程建设。实施“三旧”改造，进一步拓宽城市建设空间。加强沿江、沿湖、沿路的景观和绿道网建设，完善幼儿园、学校、医院、人防、消防、停车场等公共服务设施，建设一批功能完善、设施齐全、环境优美的住宅小区，改善市民生活环境。实施城市精细化管理，大力整治市区“脏、乱、差、堵”现象，严厉打击违建行为，规范户外广告管理，着力提升城市品位。加强县城建设，把阳春、阳西、海陵岛建成各具魅力的城市副中心。

　　以更实举措加强城镇和农村建设。深化户籍及相关配套制度改革，实施农民工积分制入户城镇等办法，引导农村人口有序向城镇聚集，促进小城镇更快发展。继续推进市级4个宜居城镇和10个宜居村庄示范点建设，建成59个县级宜居村庄示范点，创建一批名镇名村。加快城镇生活垃圾无害化处理设施建设，全面改善镇村面貌、美化镇村环境。

　　以更大力度改善生态环境。毫不动摇推进节能减排工作，大力发展低碳经济和循环经济，提高生态环境质量。加强漠阳江生态保护和综合治理，推进生态景观林、水源涵养林、水土保持林、沿海防护林、红树林等生态工程建设，强化江、湖、海水质量监测，确保水质安全。深入开展农村环境综合整治行动，启动那格五金电镀城搬迁后重金属污染整治工作。加快城北、城南等污水处理厂建设。实施南鹏岛修复、整治与保护项目。

　　六、以深化改革为动力，努力建立健全有利科学发展的体制机制

　　着力革除影响发展的体制、机制和做法，真正让有利于发展的积极性、创造性得到充分释放和激发。创新保障房建设投入、分配管理和规划建设机制，加快建立以公租房为供给主体的新型住房保障制度。不断深化医疗保障制度改革，健全统筹城乡的基本医疗保障制度。扎实推进行政管理体制改革，清理行政审批事项，全面实施并联审批，提高行政效率。认真抓好富县强镇事权改革，调整规范县市区、镇街行政事权，探索实行扁平化管理。加快事业单位人事制度改革步伐，推行全员聘用合同制和岗位聘任制。进一步理顺市、县两级财政管理体制，规范落实县市区公职人员津贴统发工作。积极探索改革投融资体制，按现代企业制度做大做优市属国有企业。稳妥实施农村综合改革，推动阳春山区县农村综合改革试点，加快农村金融发展改革，巩固集体林权制度改革成果。继续深化医药卫生、教育、文化等各项改革。

　　七、以强化公共服务为抓手，努力改善民生福利

　　建设幸福阳江是民心所向、政府之责。要更加注重保障和改善民生，把公共资源配置更多地向民生领域倾斜，扩大和改善公共服务，加快发展社会事业，集中力量办好人民群众热切期盼的实事，切实提高群众的幸福指数。

　　办好人民满意的教育事业。扎实推进中小学布局调整和规范化学校建设，确保规范化学校覆盖率达70%。加大防流控辍力度，确保初中三年保留率达94%以上。大力发展学前教育，力促全市38个镇全部建成中心幼儿园。强化高中阶段教育内涵发展，推动市第一职业技术学校和阳春中等职业技术学校创建国家级示范性中等职业技术学校。优化发展高等教育。重视发展特殊教育，推进市特殊教育学校二期扩建和县级特殊教育学校建设。加强教师队伍建设，提高师德水平和专业素质。

　　抓好人民关心的文体事业。加快广东海洋历史博物馆、市全民健身综合广场、市档案地方志党史馆等公共文体项目建设，完善市博物馆和县镇文化馆站、农民健身工程、农家书屋等公共文化体育设施。深入开展文化下乡等文化惠民活动，组织公益性文艺演出进农村、社区、企业。加强阳江历史研究，做好文化遗产的挖掘、整理、展示和保护工作。积极发展新闻传媒、文化创意、会展博览、演艺娱乐等文化产业，推动文化产业升级。

　　做好人民受惠的社会保障事业。进一步健全社会保障体系，城镇居民养老保险实现全覆盖，城乡低保实现提标扩面。高度重视“米袋子”、“菜篮子”工程建设，稳定物价总体水平。动工兴建市人民医院新住院综合大楼，推进县医院、镇卫生院标准化建设，完善区域卫生信息化平台功能。健全“惠一生”利益导向政策体系，让计生家庭优先分享经济社会发展成果。

　　今年，市政府将按照“突出重点、讲求实效、量力而行、确保落实”的原则，为群众办好以下十件民生实事。1、动用市级价格调节基金1500万元，向全市城乡低收入群众发放生活物资救助和临时价格补贴。2、全面完成扶贫开发“双到”工作任务，改造农村低收入住房困难户住房4000户。3、推进两阳中学和市高技能公共实训基地暨市技工学校三期工程建设。4、完善医疗保障体系。扩大城乡居民医疗保险覆盖面，提高财政补助标准和报销限额，城乡居民医疗保险参保率稳定在99%以上。建设完善市公共卫生医院及闸坡、平冈、城西、那龙、河口、圭岗、岗美等7个基层医疗机构。5、健全住房保障体系。在市区建设保障性住房520套。6、千方百计扩大就业。全年城镇新增就业4万人，失业人员再就业2.5万人；培训农村劳动力3万人，新增转移就业5万人；完成镇街道人力资源社会保障公共服务平台建设。7、建设特殊群体服务设施。启动市养老中心建设，建成残疾人康复中心。8、推进海堤建设。按50年一遇标准完成平冈海堤加固达标。9、改善城市环境。建设市区绿道示范段，开展马南河环境综合整治，实施市区雨污分流管网改造工程。10、改善农村生产生活条件。建成7个镇级垃圾中转站以及2525个村庄垃圾收集池，完成300公里农村公路建设。

　　八、以加强和创新社会管理为着力点，努力促进和谐稳定

　　不断加强和创新社会管理，着力在社会建设重点领域和解决突出问题上下功夫，全面推进和谐社会建设。

　　加强信访调解和应急管理。深入推进市、县、镇三级综治信访维稳机构建设。继续落实领导干部开门接访、包案处理和带案下访制度，认真排查化解矛盾纠纷。引导群众按法律程序合理表达利益诉求，从源头上预防和减少群体性事件发生。建立完善应急救援体系，不断提高应对和处置突发事件的能力。

　　解决社会突出问题。着力完善社会治安打防控体系，加强治安监控视频系统、智能交通系统和公安特警队伍建设，坚决打击黑恶势力和各种违法犯罪活动，确保治安状况持续向好。严格落实安全生产责任制和许可制度，抓好镇街安监专业队伍建设，深入开展重大危险源监控和重大隐患排查整治，严防重特大事故发生。强化食品药品监督管理，严厉打击制售假冒伪劣商品行为，规范市场秩序。加强劳动监察执法，提高劳动仲裁效能。

　　加大社区建设力度。深化基层社会管理体制改革，厘清街道办事处与社区居委会工作职责。逐步下移城乡社会管理重心，把更多资源投入到社区，让基层有权管事、有人做事、有钱办事，增强基层管理和服务能力。以社区为基本单元，完善警务区建设，落实辖区治安队、安全生产监管人员等工作职责，加强城乡社区网络化、全覆盖管理。进一步提高出租屋、流动人口和特殊人群的动态管理水平。指导各居住小区成立业主委员会，推进物业管理进社区。

　　培育新型社会组织。积极推进社会组织登记管理办法创新，放宽准入门槛，简化登记程序，为社会组织培育发展提供良好环境。重点扶持发展公益慈善类、社会服务类、群众生活类等社会组织，鼓励参与社会管理和公共服务，有效承接政府职能转移及授权委托事项。

　　同时，继续发展妇女儿童、外事侨务、民族、宗教、史志、档案、气象和防震减灾等事业，进一步做好国防教育、人民防空、民兵预备役和双拥共建活动等工作。

　　勤政廉政保发展

　　实现五年发展目标，完成今年各项任务，关键在落实，核心在于按照“实干、高效、勤政、为民、清廉”的要求，切实加强政府自身建设，全面提高政府的公信力和执行力。

　　进一步增强引领发展能力。实施大规模培训干部五年规划，不断提高各级领导干部驾驭经济社会工作、破解发展难题和做好群众工作的本领，使各级公务员勇于面对发展中遇到的各种问题，善于用新思路、新措施促进工作任务的落实。牢固树立群众观点，践行群众路线，常怀爱民之心，勤思富民之策，笃行利民之举，真心实意为民履职、帮民解忧。

　　进一步提高行政效能。加强公务员职业道德教育，倡导埋头苦干、精细务实的工作作风，营造奋勇争先、不甘人后的干事创业氛围。建立健全科学的政府绩效评估体系和经济社会发展综合评价体系，实行目标管理，加强督查考核，鞭策各级干部在其位谋其政、尽其责。加快电子政务建设，完善网络问政平台。加强各级行政服务大厅建设，所有行政审批事项在政务大厅受理、办结。进一步精简会议文件，不断提高工作效率。

　　进一步推进依法行政。认真执行人大及其常委会的各项决议、决定，定期报告工作，依法接受法律监督、工作监督。主动听取市政协的意见和建议，积极支持政协履行职能。切实做好人大代表议案、建议和政协委员提案的办理工作，健全与民主党派、工商联、无党派代表人士的联系制度。重视新闻舆论和社会公众监督，建立监察、审计、法制等部门的联合监督制度。深入开展“六五”普法教育，全面实施《阳江市人民政府依法行政五年规划》，规范行政执法行为。

　　进一步加强廉政建设。把廉政建设纳入政府领导班子、领导干部目标管理，与经济工作同部署、同检查、同考核。以决策和执行为重点环节，以人、财、物为重点领域，深化经济责任审计，加强廉政风险预警防控。教育各级领导干部带头廉洁自律，干净干事。扎实开展行风专项治理，加大纠风治乱力度，坚决纠正损害群众利益的不正之风，严肃查处各类违法违纪案件。积极开展企业评议政府部门活动，规范部门施政行为，严明工作纪律，确保政令畅通。

　　各位代表，新的起点孕育新的希望，新的征程承载新的使命。让我们在中共阳江市委的坚强领导下，团结带领全市人民，坚持以邓小平理论和“三个代表”重要思想为指导，深入贯彻落实科学发展观，以只争朝夕、奋勇当先的精神状态，以求真务实、真抓实干的工作作风，全力开创阳江科学发展的新局面，为建设幸福阳江而努力奋斗！
